# Supplementary material for: INFIMA leverages multi-omics model organism data to identify effector genes of human GWAS variants
Source: Genome Biol. 2021 Aug 23;22:241. doi: 10.1186/s13059-021-02450-8 (PMC8381555; doi:10.1186/s13059-021-02450-8)
Supplement: Supplementary file 2 — Additional file 2 Review history [file 13059_2021_2450_MOESM2_ESM.docx]

Review History

**First round of review**

**Reviewer 1**

**Were you able to assess all statistics in the manuscript, including the appropriateness of statistical tests used?** Yes: based on the report, statistical methods used seem adequate.

**Were you able to directly test the methods? No**

**Comments to author:**

The authors generate ATACseq and RNAseq data in in pancreatic islets of founder strain mice and use the resulting data to fine-map eQTLs that had been previously generated using 500 diversity outbred mice (descendants of the founder mice). They develop INFIMA to integrate the DO eqtl and the founder ATACseq and RNAseq data and provide a finer resolution of causal variants that affect transcript levels of genes. Using this finer resolution map of eQTLs, they assign effector genes to human GWAS SNPs by lifting them over to the mouse genome.

The authors generated useful molecular data and sought to assign effector genes to human GWAS risk variants, which is important and timely.

Some aspects of the paper are well written but in general I found it hard to follow. Key details and motivation for some of the definitions in the INFIMA method are missing, making it very hard to understand. Rewriting several sections to make it more accessible to readers less familiar with mouse genetics would be helpful.

The restriction to local-ATAC-QTL markers seems limiting. Given the small sample size in founder data, the power to detect ATAC-QTLs is limited. I would think that anchoring the mouse annotation to DO eQTLs but with improved credible sets using with the founder ATAC-seq and RNAseq data as priors would be more fruitful but this may be more of a philosophical difference in approach between the authors and this reviewer. The methods I would recommend for this would be one of the more recent fine-mapping approaches such as DAPG or SUSIER. Nevertheless, there is a huge amount of work presented in this paper and the enrichment of 3D contact evidence suggests that INFIMA is identifying relevant effector genes.

Below is a list of my comments to the authors:

--------------------------------------------------------

The shiny application is very helpful but the authors should also make the full set of results publicly available (in zenodo or other repositories with permanent records and DOI).

It's good to see that the code has been made available in Github. I would encourage the authors to make available the data (or intermediate results) in an easily reproducible format accompanying the code.

Comparison with other approaches:

To me, a more straightforward approach would be to apply the recently developed fine-mapping methods such as DAPG or SUSIER to the DO eQTL data using the founder omic data as priors. But I realize that this may be beyond the scope of this paper.

When you say causal local-ATAC-QTL, do you mean causal for transcript levels? It could also mean causal for ATACseq peak. Or are you implicitly assume that the local-ATAC-QTLs you find are causal for the peaks? More consistent definition and use of terminology would make this paper much easier to read.

pg 2 line 19 says: "However, these [TWAS] approaches do not link the effector genes to SNPs". It would be more accurate to say that "However, these approaches do not directly link the effector genes to individual SNPs". The way it is stated the sentence is somewhat misleading. While it is true that TWAS methods do not directly assign effector genes given a genetic variant, they do assign effector genes to the GWAS locus. This assignment is done implicitly, since significant genes in TWAS will have significant variants as genetic predictors.

Fig 4b shows the number of top local-ATAC-QTL associations with the allele patterns. There is no mention on how that was generated and thus unclear how to interpret what's shown. What's the definition of allele pattern? Maybe obvious to mouse geneticists?

INFIMA model: I found the the description of the model extremely hard to follow.

The motivation of some of the modeling decisions (like the distance between DO allelic effects and founder allelic effects) are unclear and maybe because of that they seem unnecessarily complicated. Also there seem to be a number of inaccurately described variables making it even harder to follow the model construction.

Bg is defined as the normalized expression of gene g and it's a vector of length p_g. Shouldn't it be a vector of length 8., one for each founder strain?

From figure 1, allele affects vary by genomic position. How is this summarized in the Y vector (8x1), which has only one value for each strain within the whole window of size W?

Pg 7 line 36n Unclear what this means: "it already enables multiple causal loci per gene when the gene is associated with multiple markers"  Does it mean that different windows could be overlapping and thus many markers analyzed multiple times?

pg 7 line 52: you mention allelic effects of chromatic accessibility. I don't think you are using this in this paper. If you do, you may want to make it more clear.

pg 8 line 11: Unclear to me why "However, some concordance between DO mice allelic expression Yg and founder gene expression Bg is still

warranted." This may make sense but more explanation is needed. For example, using the similarity between allelic effects by strain (Yg) with the expression level might make sense, since that could be capturing the effect of causal variants nearby not captured by the restricted set of local-ATAC-QTL SNPs. Some discussion of this would help understand some of the decisions made in the INFIMA model definition.

edit distance model: why not use the more natural difference between quantitative traits as distance measure? You explain that you don't want to introduce additional parameters. In fact, the additional parameters are being introduced by your definition of differences. How do you decide on what's lower, the same, higher.

In general by trinarizing you lose power. If you must trinarize for some reason, why not trinarize the differences rather than take differences of trinarized variables. You may need to standardize Eg, and Bg, but that can be done in the quantitative scale.

Why use multinomial distribution of the differences between Yg and Eg or Bg? You don't make use the natural ordering of the differences coded as 0, 1, and 2.

Vg is boldfaces in eq (1). Is this the same as the not bf Vg? If so, please keep format consistent.

How is Eg calculated from the founder RNAseq data. The method states that you use matrixeQTL but that wouldn't give you strain specific allelic effects. Are you using allele specific expression?

pg 10 line 9: "The apparent bimodal distribution indicates that INFIMA infers a causal local-ATAC-QTL for 3,846 (38.0%) DO-eQTL genes at FDR of 0.05." Why the bimodal distribution is relevant for the conclusion here? If there are 3,846 genes with high posterior probability of causal would suffice.

The restriction to local-ATAC-QTL allows you to reduce the search space of causal SNPs but it also may mislead you. It is possible that this approch takes you closer to the causal variant, even if it's not within the ATACseq peak or did not get identified by the ATAC-QTL association.

The authors used matrixQTL for the association analysis of founder local-ATAC-QTLs and gene expression. A method such as RASQUAL or WASP which use allele specific reads rather than total read counts would be more appropriate.

**Reviewer 2**

**Were you able to assess all statistics in the manuscript, including the appropriateness of statistical tests used?** Yes

**Were you able to directly test the methods?** No

**Comments to author:**

This manuscript presents a statistical integration framework that aims to fine map mouse expression quantitative loci and leverage these results to infer candidate susceptibility genes for human diabetes GWAS SNPs. There is certainly great current interest in both fine mapping risk variants and linking these variants to genes.

In brief, the authors filter existing mouse eQTL data from islets via chromatin accessibility data (ATAC-seq), and then attempt to resolve further to transcription factor binding sites. This is a standard approach to narrow likely causal variants. Further, they attempt to filter by variants associated with local chromatin accessibility "ATAC QTLs". In essence, what they are trying to accomplish is to prioritize variants by nonrandom associations with peaks and transcription factor footprints. This is just to gather evidence for a functional variant, then use their model to integrate signals, some of which are independent and some not. Validation studies are applied via simulation and comparison with chromatin interaction data. Translation to the human genome and human disease is attempted by resolving SNPs and genomic features between the human and mouse genome.

Overall, I found the paper to be highly confusing and to apply what I would consider to be many non-standard approaches, and also to lack transparency in the description of some methods and figures. The INFIMA model itself is not unreasonable, but the substrates on which it is applied, particularly the ATAC-QTL data, render the output questionable. The translation to human studies is problematic.

My chief concerns can be summarized as follows:

1. ATAC data from islets. The total number of peaks they identify is quite low, and there is no indication of the quality of the data (TSS enrichment score).

2. ATAC QTLs. This is a major weakness of the paper. 'QTLs' is a misnomer, and this is highly problematic.

2.1 'QTL' is a misnomer for the approach they apply. With only a handful of outbred lines, and particularly with a lack of heterozygotes, they do not have either the variants or the numbers to call true QTLs. GIven the lack of heterozygotes, they cannot exploit allelic imbalance. Rather, what they are doing is a permutation test and calling it a QTL. Under the circumstances, their approach is not unreasonable -- but it is definitely NOT a QTL.

2.2 The authors identify an absolutely enormous number of "local ATAC QTLs" -- 47,000 in only 22,000 peaks. This number is approximately 10X the rate that others have observed for variants that alter chromatin accessibility. It cannot be correct.

3. TF footprints are central to their analysis but the handling of these is problematic in many respects.

3.1 There is no evidence that the depth metric the authors employ is actually meaningful, since there does not appear to be any correction applied for the numerous biases and complexities inherent in ATAC-seq insertions. This requires validation.

4. The above problem with 'ATAC-QTLs' pervades the differential transcriptome analysis.

4.1 The conclusion that "a strong contribution of genetic variance of chromatin architecture within promoters to proximal gene regulation" is practically a platitude, having been amply demonstrated in a very large number of studies over the past decade or more.

5. The INFIMA model is not unreasonable, but it is extremely densely and confusingly presented. Some clear diagrams indicating the workflow and critical dependencies would help the reader understand it more clearly.

5.1 I am concerned that the simulations employed to validate the results may be overfitting to the relatively small data sets.

6. The authors presume that chromatin interactions via chromatin conformation capture are a gold standard for linking elements to genes. This is far from the case. What 'C' assays measure are proximity, and they are generally blind within 10-20kb of a TSS where. There is no indication how many variants fall into this zone.

6.1 Even apart from the issue with ATAC-QTLs the claim that "INFIMA prediction of local-ATAC-QTLs for DO-eQTL genes tend to be supported by higher Hi-C interaction signal" is very weak. The significance is achieved via a highly sensitive distribution statistic (K-S); the fact that there are significant -- but quantitatively small -- differences in the aggregate is not convincing.

7. The translation to human studies -- and hence support for the claim that target genes of GWAS studies can be assigned by their approach -- is highly problematic.

7.1 A key difficulty is presented by the translation of GWAS SNPs to the mouse genome. The overwhelming majority of GWAS SNP positions (80%) cannot be resolved directly in the mouse genome. This is expected. However, what the authors then attempt to do is to resolve these to peaks in the human genome, then resolve these peaks into the mouse genome. A priori one would have again expected a small number of correspondences. But they authors make an additional step, which not to resolve the peaks directly but to approximate them by assigning to the nearest mouse peak within 10kb. This is completely arbitrary, and not scientifically justified.

7.2 Associations between SNPs and human genes are conditioned on the above, and thus of concern.

8. Fig S18 suggests that Cast is driving much of their signal -- which highlights the fact that strain biases are not well accounted for.

8.1 It is also not clear to me how one can get a negative abs log p value; how does one understand a p value greater than 1?

9. On a general level, I found many of the figures to be confusing, lacking adequate explanation in the legends. Virtually none of them were what I would consider self-explanatory.

**Authors Response**

We are grateful to the reviewers for their constructive comments that improved our manuscript. In what follows, both reviewers’ comments are itemized in the order they were presented. We respond to these individually by providing additional data, calculation, or clarifying explanations. We also included the changes made in the manuscript in response to each reviewer comment. As suggested by two reviewers, we no longer use “local-ATAC-QTLs” to avoid confusion with the use of chromatin QTL (cQTL) and, instead, we refer to local ATAC-signal modulating variants as “local-ATAC-MV”.

**Response to Reviewer #1**

*1.“The shiny application is very helpful but the authors should also make the full set of results publicly available (in zenodo or other repositories with permanent records and DOI). It's good to see that the code has been made available in Github. I would encourage the authors to make available the data (or intermediate results) in an easily reproducible format accompanying the code.”*

**Response:**

We thank the reviewer for reviewing our INFIMA R package as well as the shiny app. We have now created a permanent link for the shiny application <http://www.statlab.wisc.edu/shiny/INFIMA/>.

In addition, we have uploaded the processed data and the results to a Zenodo repository with DOI 10.5281/zenodo.4625293. The source code for reproducing the results is available at <https://github.com/keleslab/INFIMA-paper>.

*2.“Comparison with other approaches:*

*To me, a more straightforward approach would be to apply the recently developed fine-mapping methods such as DAPG or SUSIER to the DO eQTL data using the founder omic data as priors. But I realize that this may be beyond the scope of this paper.”*

**Response:**

We thank the reviewer for raising this issue, which motivated us to more formally benchmark the performances of human GWAS fine-mapping methods on data collected from model organisms; e.g., DO mice. Our initial applications of human GWAS fine-mapping methods on DO-eQTL were hindered by the high LD of the DO mice data. A direct application of the fine-mapping methods to DO-eQTL results in considering all the SNPs around the marker as candidates. For example, at the *Adcy5* locus, there are 27,307 SNPs within 1 Mb distance to the marker of *Adcy5* and we observe an excessive number of perfect LD cases (Response Fig. 1). When we test DAP-G and SuSiE for the *Adcy5* locus with such a larger number of putative causal SNPs (27,307), both approaches fail. The top signal cluster estimated by DAP-G contained 3,590 SNPs with cluster Posterior Inclusive Probability (PIP) of 0.434 and average LD of 0.956. SuSiE did not output any credible sets for any coverage level. The maximum estimated SNP PIPs for both approaches were <0.003. Next, as suggested by the reviewer and to reduce the size of the credible sets, we also tried an informative prior for the 27,307 SNPs based on 3 categories: (i) not within an ATAC-seq peak; (ii) within an ATAC-seq peak but not local-ATAC-MV; (iii) identical to local-ATAC-MV. For (iii), we utilized the INFIMA multi-omics priors. However, the outputs from DAP-G and SuSiE were almost identical with or without using a prior. We observed similar and even further computational issues with PAINTOR, CAVIARBF, and FINEMAP. We have now added the *Adcy5* locus as a showcase of how standard fine-mapping applications designed for human genetic studies do not perform well on genetic data collected from DO mice.

Next, we considered two additional computational experiments and used the reduced set of local-ATAC-MVs as input SNPs with and without the multi-omics prior and evaluated their performances as we have done for INFIMA (as in the Fig. 7c of the main paper). We note that the 8 founder mice are homozygous yielding only 2^7 = 128 different genotypes of the local-ATAC-MVs (B6 is a reference strain). As a result, perfect LD issues are also abundant for INFIMA, DAP-G, and SuSiE (Response Fig. 2). The multi-omics priors (distance, footprint annotations, concordance between ATAC-seq and RNA-seq) have the potential to break the ties. Like Fig. 7c, we benchmarked the performances of multiple fine-mapping strategies with empirical cumulative distribution of normalized Hi-C scores (Response Fig. 3). Without prior, DAP-G and SuSiE are better than “Random” but worse than "Closest to Marker". With multi-omics data prior, as expected, performances of DAP-G and SuSiE improved markedly but were still worse than "Closest to Gene". So, notably, DAP-G and SuSiE with multi-omics data priors do not perform better than the simple baseline approaches in the DO mice setting. Both DAP-G and SuSiE are inferior to the performance of INFIMA “Most Likely” predictions.

In summary, while recent fine-mapping methods like DAP-G and SuSiE exhibit promising performances in human GWAS, they fail due to excessive high LD in the DO mice setting. Furthermore, with a reduced set of SNPs and priors based on multi-omics data, DAP-G and SuSiE are still inferior to INFIMA and simple baseline approaches. In contrast, INFIMA model aims to capture the multi-omics data generative processes between DO and founder mice, which is likely the key factor for INFIMA’s better performance.

Response Fig. 1: The DO mice LD matrix of the imputed SNPs at the *Adcy5* locus. Rows and columns depict the 4,616 SNPs within the 200 Kb of the eQTL marker and are ordered with respect to their genomic coordinates. Red color indicates a perfect LD.

Response Fig. 2: The DO mice LD matrix for the 3,671 local-ATAC-MVs from chromosome 1. Rows and columns depict local-ATAC-MVs and are ordered with respect to their genomic coordinates. Red color indicates a perfect LD.

Response Fig. 3: Evaluation of fine-mapping strategies with empirical cumulative distribution of normalized easy Hi-C scores. “Most Likely” and “Least Likely” refer to the most and least likely predictions from INFIMA, respectively.

*3.“When you say causal local-ATAC-QTL, do you mean causal for transcript levels? It could also mean causal for ATACseq peak. Or are you implicitly assume that the local-ATAC-QTLs you find are causal for the peaks? More consistent definition and use of terminology would make this paper much easier to read.”*

**Response:**

To avoid this confusion, we now refer to “local-ATAC-MV” which stands for “local ATAC-seq signal modulating variant”. Here, the notion of the modulating variant is aimed to capture the variability of the ATAC-seq signal that can be explained by the local variants, i.e., variants within the peak.

We have now paid extra attention to the use of “causal local-ATAC-MV” throughout the manuscript. The first occurrence of this term is on page 7 line 32, and appears as: “INFIMA model assumes at most one causal local-ATAC-MV per gene for a single marker-gene association”. Here, “causal local-ATAC-MV indicates that the local-ATAC-MV is causal for the expression level of a DO-eQTL gene. We mean the same for all later occurrences of “causal local-ATAC-MV”.

*4.“pg 2 line 19 says: "However, these [TWAS] approaches do not link the effector genes to SNPs". It would be more accurate to say that "However, these approaches do not directly link the effector genes to individual SNPs". The way it is stated the sentence is somewhat misleading. While it is true that TWAS methods do not directly assign effector genes given a genetic variant, they do assign effector genes to the GWAS locus. This assignment is done implicitly, since significant genes in TWAS will have significant variants as genetic predictors.”*

**Response:**

Many thanks for these suggestions and explanations. We have modified the statement about TWAS as suggested to make it more accurate.

*5.“Fig 4b shows the number of top local-ATAC-QTL associations with the allele patterns. There is no mention on how that was generated and thus unclear how to interpret what's shown. What's the definition of allele pattern? Maybe obvious to mouse geneticists?”*

**Response:**

We apologize for not clearly explaining the terms “allele pattern” or “allelic pattern”. Since the founder mice are homozygous, “allele pattern” for them exactly corresponds to their “genotype. We have changed all occurrences of “allele pattern” into “genotype” when allele pattern coincided with genotype.

To avoid confusion, we have modified the caption and the y-axis label of Fig. 4b. New caption: “b. UpSet plot for the frequencies of **genotypes** of local-ATAC-MVs associated with founder islet gene expression. … . **Genotypes** with frequencies less than 50 are not displayed.” New y-axis label: “Frequency of genotype of top local-ATAC-MVs associated with genes”.

We associated local-ATAC-MVs with gene expressions using MatrixEQTL (Shabalin, *Bioinformatics*, 2012). For each gene, we picked its best associating local-ATAC-MV and summarized its genotype. The frequencies of genotypes of the top local-ATAC-MVs associated with genes are shown in Fig. 4b. The main objective of Fig. 4b is to illustrate that gene expression patterns are largely driven by CAST, PWK, and WSB alleles. We edited the text to make these points more clear.

*“6.INFIMA model: I found the the description of the model extremely hard to follow.*

*The motivation of some of the modeling decisions (like the distance between DO allelic effects and founder allelic effects) are unclear and maybe because of that they seem unnecessarily complicated. Also there seem to be a number of inaccurately described variables making it even harder to follow the model construction.”*

**Response:**

We apologize for not describing the INFIMA model clearly. We have revised the section “INFIMA model for fine-mapping DO mouse islet eQTLs by leveraging founder strain islet ATAC-seq and RNA-seq” for clarity. In addition, we designed an example to illustrate each piece of the input data as well as the edit distances with multinomial distributions (Response Fig. 4). Response Fig. 4 is also included in the main Fig. 5 in the revised manuscript.

Response Fig. 4: An example input of the INFIMA model. **(a).** An overview of a 1 Mb window around a DO-eQTL marker (centered dashed line) associated with *Gene1*. Two out of five candidate local-ATAC-MVs (red short lines) are decorated with footprint annotations (orange triangles). **(b).** Example input data for the five candidate local-ATAC-MVs. A_g: local normalized ATAC-seq peak signal; F_g: footprint annotation indicator; D_g: distance score; E_g: founder RNA-seq genotype effect sizes, non-zero entries mean alternative alleles; B_g: normalized founder RNA-seq gene expression; Y_g: the allelic effects for the DO-eQTL with respect to *Gene1*. **(c).** An illustration of data trinarization and edit distance with multinomial distributions. Z_g: hidden indicator of causal candidate local-ATAC-MV. \tilde E_g (\tilde R_g is one row of \tilde E_g), \tilde Y_g, and \tilde B_g are trinarized data of E_g, Y_g, and B_g, respectively. The trinarization details can be found in Methods. **(d).** The edit distance variables (assumed to follow multinomial distributions) are obtained by counting how many strains have 0, 1, or 2 absolute distances between \tilde Y_g and \tilde R_g/B_g.

*“7.Bg is defined as the normalized expression of gene g and it's a vector of length p_g. Shouldn't it be a vector of length 8., one for each founder strain?”*

**Response:**

We apologize for this typo. B_g, as a normalized expression of gene g across 8 founder strains, should be a vector of length 8. We have corrected the typo in the manuscript.

*“8.From figure 1, allele affects vary by genomic position. How is this summarized in the Y vector (8x1), which has only one value for each strain within the whole window of size W?”*

**Response:**

Fig. 1 illustrates the Diversity Outbred (DO) mice eQTL analysis results at the *Abcc8* locus. The DO-eQTL data was generated and analyzed in another paper (Keller *et al.*, *Genetics*, 2018), where the marker that maximizes the LOD score based on R/qtl2 (Broman *et al.*, *Genetics*, 2019) was chosen as eQTL at each locus. For the *Abcc8* gene, the marker chr7:46,000,542 with the highest LOD score of 64.58 was selected as the eQTL marker. The corresponding 8x1 Y vector [-0.396, 0.327, 0.605, 0.306, 0.601, 0.551, -1.970, -0.024] summarizes these estimated allele effects. We have uploaded the DO-eQTL data to Zenodo as well.

*“9.Pg 7 line 36n Unclear what this means: "it already enables multiple causal loci per gene when the gene is associated with multiple markers"*

*Does it mean that different windows could be overlapping and thus many markers analyzed multiple times?”*

**Response:**

The reviewer’s interpretation is correct. For the DO mice eQTL data, one DO gene can have multiple markers within its 1Mb window. As a consequence, the same gene with multiple eQTL markers can be analyzed multiple times with overlapping windows. This allows the possibility of multiple causal SNPs.

*“10.pg 7 line 52: you mention allelic effects of chromatic accessibility. I don't think you are using this in this paper. If you do, you may want to make it more clear.”*

**Response:**

We apologize for the excessive usage of the term “allelic effect”. We have revised the whole section. To be more precise, we mean the alleles from different strains may have impact on both gene expression and chromatin accessibility. Our ATAC-seq is from homozygous founders. Thus, genotype effect is equivalent to allelic effect. To make the manuscript clearer, we have changed “Specifically, we convert quantitative allelic effects across strains (i.e., expression and chromatin accessibility) to trinary indicators” to “Specifically, we convert Y_g, R_g, and B_g to trinary indicators”.

*“11.pg 8 line 11: Unclear to me why "However, some concordance between DO mice allelic expression Yg and founder gene expression Bg is still*

*warranted." This may make sense but more explanation is needed. For example, using the similarity between allelic effects by strain (Yg) with the expression level might make sense, since that could be capturing the effect of causal variants nearby not captured by the restricted set of local-ATAC-QTL SNPs. Some discussion of this would help understand some of the decisions made in the INFIMA model definition.”*

**Response:**

We totally agree with the reviewer. When there is no causal local-ATAC-MV, it is possible that some SNPs beyond the set of local-ATAC-MVs are causal. DO mice are obtained by outcrossing 8 founder mice strains. We would still expect some similarity between DO gene expressions and founder gene expressions. This is why we utilize the Null generative model to incorporate such cases.

*“12...edit distance model: why not use the more natural difference between quantitative traits as distance measure? You explain that you don't want to introduce additional parameters. In fact, the additional parameters are being introduced by your definition of differences. How do you decide on what's lower, the same, higher.*

*In general by trinarizing you lose power. If you must trinarize for some reason, why not trinarize the differences rather than take differences of trinarized variables. You may need to standardize Eg, and Bg, but that can be done in the quantitative scale.”*

**Response:**

We would like to thank the reviewer for this great question on modeling choices. We acknowledge that given this data, there might be other formulations of the problem. Our overarching goal was to capture trends in a way that improves the interpretation, without focusing on quantifying the actual quantitative differences.

We performed trinarization on Y_g (DO allelic effect), B_g (normalized founder RNA-seq gene expression), and E_g (founder RNA-seq genotype effect, R_g = E_g^T Z_g is a row of E_g). After this, we modeled the absolute differences between trinarized Y_g and R_g/B_g using multinomial distributions. The reasons why we trinarized and utilized multinomial distributions can be summarized as follows:

1. Why do we trinarize? As B6 is the reference mouse strain, it is natural for a mouse geneticist to ask how another strain (e.g. CAST) compares to B6 in terms of allelic effects and expression of genes. Instead of modeling the quantitative natural differences which we had considered as an alternative model, we were more interested in the trends, i.e. high, low, or the same level as B6. The cutoffs for trinarization was selected based on quantiles of the standard distribution while balancing the number of cases in the three levels {-1, 0, 1} of Y_g, R_g, and B_g (Methods - Trinarization of allelic expression effect sizes into allelic patterns).
2. Why do we choose multinomials? There are likely other plausible models. An alternative model that we have considered is a mixture of point mass at zero and multiple normal distributions. Specifically, as also suggested by the reviewer, we standardized Y_g and B_g/R_g to [0, 1] and calculated their natural differences . By construction, the minimum/maximum is coded as 0/1. When Y_g and B_g/R_g agree on the minimums/maximums, their natural differences are zero, giving rise to a point mass at zero. The distribution of the rest of the natural differences between standardized Y_g and B_g appeared to be composed of more than 3 normal mixtures (Response Fig. 5), implying at least 7 parameters (1 for point mass at zero, 3 for means, and 3 for variances) for this mixture model. In contrast, our multinomials require only 4 parameters (2 for normal strains, 2 for wild strains). Moreover, the founder ATAC-seq and RNA-seq data have illustrated that wild strains CAST, PWK, and WSB are quite different from normal strains. Therefore, a unified model with separate parameters for both wild strains and normal strains should be more preferable. Multinomial distributions are good choices in this sense. In addition, multinomials offer an easy way to incorporate priors with a conjugate Diricihlet prior. However, not having fitted this specific mixture model, we acknowledge that we can’t say a priori whether there will be significant differences in the inference.
3. Does trinarizing before or after taking differences matter a lot? As we have discussed above, the main reason for trinarization is to model the trends in strains compared to B6. Trinarizing before taking differences directly follows our purpose of modelling such trends. The point of using edit distance random vectors is to measure the degree of discrepancy between Y_g and R_g/B_g coded in {0, 1, 2} as illustrated in Response Fig. 4c-d. We obtain edit distances with 56.2% 0’s, 36.8% 1’s, and 7.0% 2’s by trinarizing Y_g and B_g before taking differences. If we trinarize afterwards, the distributions of {0, 1, 2} are not very far off from those trinarized before taking the differences. Response Fig. 6 illustrates that the edit distances from trinarizing before and after taking differences are largely similar. Some perturbations in distances are expected by definition. In addition, we do not observe any large contrasts (before = 0 and after = 2, before = 2 and after = 0) in edit distances.

Response Fig. 5: Normal mixture modeling of natural distances. **(a)** The distribution of the natural difference between standardized Y_g and standardized B_g excluding the zero point mass (standardized to [0, 1] closed interval). **(b)** The Bayesian Information Criterion (y-axis) vs. the number of normal mixture components (x-axis). “E” and “V” stand for equal variance and unequal variance normal mixtures. The results were obtained by using the R package mclust.

Response Fig. 6: A comparison between trinarization before and after taking differences. There are 10,396 (# of DO eQTL marker - gene cases) by 8 (strains) edit distances in total. The number shown in the matrix entry [i, j] is the number of edit distances fall into the corresponding category. The color of [i, j] is the proportion of the edit distances with respect to column sums.

*“13.Why use multinomial distribution of the differences between Yg and Eg or Bg? You don't make use the natural ordering of the differences coded as 0, 1, and 2.”*

**Response:**

After trinarization, the edit distance, i.e., the absolute difference between Y_g and E_g or B_g, can be 0, 1, or 2. Multinomial distributions are straightforward when we aim to assign a likelihood for an observed edit distance. The key issue with multinomials, as mentioned by the reviewer, is that the categories are nominal. Intuitively, smaller distances should be favored with a higher likelihood. When we estimated the parameters of multinomials, we had closed form MLEs (Equations (23), (24)). We then enforced the natural ordering of absolute distances 0, 1, and 2 by introducing bias (pseudocounts) towards smaller absolute distances (Equations (27) - (30)). In summary, we biased the MLEs for multinomial distributions to account for the natural ordering of absolute differences.

*“14.Vg is boldfaces in eq (1). Is this the same as the not bf Vg? If so, please keep format consistent.”*

**Response:**

Many thanks for catching this typo. V_g should never be in boldface. We have corrected the Equation (1).

*“15. How is Eg calculated from the founder RNAseq data. The method states that you use matrixeQTL but that wouldn't give you strain specific allelic effects. Are you using allele specific expression?”*

**Response:**

We apologize again for the confusing effect of using genotype effect and allelic effect interchangeably. We simply performed marginal regression of B_g (the founder expression of gene g) on the genotype of candidate SNPs. The idea is identical to MatrixEQTL and allele specific expression data is not applicable in our setting. We refer to the regression coefficient, i.e. the effect size, as the founder RNA-seq genotype effects. Imagine a p_g by 8 genotype matrix coded with 0 and 2’s (homozygous samples) with all of the “2’s” replaced by the corresponding effect sizes. The modified genotype matrix with these effect sizes from marginal regression gives us E_g. Please refer to Response Fig. 4b to see what a typical E_g looks like.

We clarified the definition of E_g by modifying page 7 line 28-30 to “E_g is a p_g by 8 matrix of founder RNA-seq genotype effects of these candidate SNPs for gene g (i.e. marginal regression of gene expression with respect to genotype).” In addition, we modified page 19 line 60-62 accordingly to explain how we trinarized E_g and R_g.

*“16. pg 10 line 9: "The apparent bimodal distribution indicates that INFIMA infers a causal local-ATAC-QTL for 3,846 (38.0%) DO-eQTL genes at FDR of 0.05." Why the bimodal distribution is relevant for the conclusion here? If there are 3,846 genes with high posterior probability of causal would suffice.”*

**Response:**

Thanks for pointing out that the bimodal distribution is irrelevant for the conclusion. We have changed this sentence into “It indicates that INFIMA infers a causal local-ATAC-MV for 3,846 (38.0%) DO-eQTL genes at FDR of 0.05”.

*“17.The restriction to local-ATAC-QTL allows you to reduce the search space of causal SNPs but it also may mislead you. It is possible that this approch takes you closer to the causal variant, even if it's not within the ATACseq peak or did not get identified by the ATAC-QTL association.”*

**Response:**

We totally agree with the reviewer’s comments and acknowledge the limitations of this framework. It is possible that the real causal SNP is beyond set implied by the local-ATAC-MVs, which is the reason why we introduce the hidden variable V_g to identify such cases (the indicator for if the DO eQTL gene contains a causal SNP among the candidate local-ATAC-MVs).

INFIMA model allows for such possibility with the “Null/Causal generative model” given V_g = 0/1, respectively. Under the current model setting, we are able to estimate the posterior probability V_g and identify the cutoff of V_g at FDR of 0.05. It turned out that we kept 38% of DO-eQTL genes at FDR of 0.05. The remaining 62% of genes, that we couldn’t explain through SNPs, certainly fell into the category that the causal SNP is beyond the set of local-ATAC-MVs. There is room to improve our INFIMA model in the future by considering candidate SNPs beyond local-ATAC-MVs. Nevertheless, local-ATAC-MVs provide direct biological insights in terms of TF binding in open chromatin. It may not be straightforward to link SNPs outside ATAC-seq peaks, but it could be a promising extension for the INFIMA model especially as we collect additional genomic data (e.g., histone ChIP-seq, eCLIP-seq for RNA binding proteins and so on) on founder strains. We have included more discussion on this point in our revised manuscript.

*“18. The authors used matrixQTL for the association analysis of founder local-ATAC-QTLs and gene expression. A method such as RASQUAL or WASP which use allele specific reads rather than total read counts would be more appropriate.”*

**Response:**

The founder mice that are used to derive founder local-ATAC-MVs (previously referred to as *local-ATAC-QTLs)* are homozygous. As a result, parent of origin calculations enabled by RASQUAL or WASP are not applicable for founder strains. MatrixEQTL enables assessing the associations between founder local-ATAC-MVs and gene expressions in a high throughput and computationally efficient fashion.

**Response to Reviewer #2**

*“1. ATAC data from islets. The total number of peaks they identify is quite low, and there is no indication of the quality of the data (TSS enrichment score).”*

**Response:**

We thank the reviewer for emphasizing the importance of TSS enrichment results as a QC metric for ATAC-seq peaks. We had followed the ENCODE ATAC-seq processing pipeline strictly before we performed any subsequent downstream analyses. We made sure that our peaks were reproducible according to best practices and established criteria in the field. Our founder mice ATAC-seq data had passed the quality control according to ENCODE standards <https://www.encodeproject.org/atac-seq/#standards>. As part of our revision, we performed TSS enrichment analysis using ataqv from the Parker Lab at the University of Michigan (Response Fig. 7). 15 out of 16 ATAC-seq samples have TSS score > 15, which is classified as “ideal” quality according to ENCODE. The TSS score of the 129 female sample is 14.09, which is close to the “ideal” category and well beyond the “acceptable” cutoff. We also included the TSS enrichment results in Additional file 1.

We would like to further elaborate on the merging of peaks across samples. After peak-calling for each ATAC-seq sample at FDR of 0.05, we utilized IDR (Irreproducible Discovery Rate: which is a standard method established by the ENCODE project for analysis of ChIP-seq, ATAC-seq, DNase-seq, eCLIP-seq datasets) to generate concordant peaks between replicates. The final numbers of peaks in each IDR peak file are summarized in the Response Table 1. We have 7 out of 8 IDR peak files with > 70,000 peaks, classified as “ideal” quality according to ENCODE. The IDR peak file for AJ strain contains 64,684 peaks, which is still well above the “acceptable” cutoff of 50,000 peaks. We then merged IDR peaks to obtain 92,570 master peaks. Next, we applied quality trimming (see Methods - ATAC-seq data analysis as well as Additional File 1) and reduced the number to 51,014. In summary, our numbers of peaks are very comparable to high quality studies. In fact, the numbers of IDR thresholded ATAC-seq peaks from mouse liver (ENCSR255XTC), stomach (ENCSR363SKQ), kidney (ENCSR758IRM), from ENCODE are 55,222, 53,843, and 54,619 respectively.

Response Fig. 7: Summary of TSS enrichment analysis.

Response Table 1: Summary of the number of ATAC-seq peaks for each sample and the IDR peaks.

*“2. ATAC QTLs. This is a major weakness of the paper. 'QTLs' is a misnomer, and this is highly problematic.*

*2.1 'QTL' is a misnomer for the approach they apply. With only a handful of outbred lines, and particularly with a lack of heterozygotes, they do not have either the variants or the numbers to call true QTLs. GIven the lack of heterozygotes, they cannot exploit allelic imbalance. Rather, what they are doing is a permutation test and calling it a QTL. Under the circumstances, their approach is not unreasonable -- but it is definitely NOT a QTL.*

*2.2 The authors identify an absolutely enormous number of "local ATAC QTLs" -- 47,000 in only 22,000 peaks. This number is approximately 10X the rate that others have observed for variants that alter chromatin accessibility. It cannot be correct.”*

**Response to (2), (2.1), and (2.2):**

We completely agree with ‘QTL’ being a misnomer for our setting where we have homozygous genotypes and we are simply quantifying association between genotypes and accessibility. We have now changed the name to local-ATAC-MV, i.e. **local ATAC**-seq peak **M**odulating **V**ariant.

We also agree with the reviewer that 47,062 SNPs in 22,200 peaks seem enormous. This is due to the correlation structure of the SNPs in the founder strains. With 8 founder mice, the genotype space, i.e., values that the vector of genotypes can take, contains only 2^7 = 128 different values (B6 is always a 0). Although we started with millions of founder SNPs and managed to narrow down the number, peaks harbor groups of SNPs with the exact same genotypes across the strains (Response Fig. 8). In fact, the effective number of local-ATAC-MVs (counting the SNPs within the same ATAC-seq peak and the same genotype across the founder strains only once) is 23,760. This is a number more comparable to chromatin QTLs from human studies (e.g., 2,450 to 22,624 chromatin QTLs from LCLs Grubert *et al*. (*Cell*, 2015)).

Response Fig. 8: The correlation matrix for chr1 local-ATAC-MVs. Red color indicates a perfect correlation.

*“3. TF footprints are central to their analysis but the handling of these is problematic in many respects.*

*3.1 There is no evidence that the depth metric the authors employ is actually meaningful, since there does not appear to be any correction applied for the numerous biases and complexities inherent in ATAC-seq insertions. This requires validation.”*

**Response to (3) and (3.1):**

We appreciated these concerns related to TF footprinting analysis. Our manuscript utilizes footprint signals in two separate cases ((i) Fig. 3a; (ii) Fig. 3c). In both cases, we utilized existing approaches.

(i) Specifically, Fig. 3a leverages a widely used method PIQ (Sherwood *et al*., *Nature Biotechnology*, 2014). PIQ models footprints with Gaussian processes and uses expectation propagation to compute footprints in a motif-specific manner and adjust for motif specific backgrounds. A similar strategy was also used by Yardimci *et al.*, (*Nucleic Acids Research*, 2014) and Karabacak Calviello *et al.*, (*Genome Biology*, 2019) to adjust for sequence specific biases by considering footprint (i.e., motif)-specific backgrounds. In addition, an in depth comparison of different footprint analysis methods (Gusmao *et al*., *Nature Methods*, 2016) established PIQ as one of the top three footprint analysis methods. Furthermore, our results from PIQ are enriched with footprints of known islet-specific TFs, providing biological support.

(ii) The analysis in Fig. 3c elucidates the impact of SNPs on footprints. Because the exact same genomic region is compared across strains with the reference and alternative alleles, this analysis is insensitive to potential sequence-specific biases. In addition, this analysis uses the concept of footprint depth (FPD) from Baek *et al.* (*Cell reports*, 2017) in a way that accounts for local biases around the footprint (FPD, Additional file 1: Fig. S20).

*“4. The above problem with 'ATAC-QTLs' pervades the differential transcriptome analysis.”*

**Response:**

We acknowledge the misnomer in using “QTL” for SNPs, genotypes of which are associated with the ATAC-signal. The differential transcriptome analysis identifies local-ATAC-MVs, genotypes of which associate with transcriptomic differences of the strains. Perhaps more importantly, these calculations ultimately serve as dimension reduction for the key task of fine-mapping the DO mice eQTLs.

*“4.1 The conclusion that "a strong contribution of genetic variance of chromatin architecture within promoters to proximal gene regulation" is practically a platitude, having been amply demonstrated in a very large number of studies over the past decade or more.”*

**Response:**

Thank you for the comment. We have changed “a strong contribution of genetic variance of chromatin architecture within promoters to proximal gene regulation” into “a strong contribution of genetic variance of chromatin architecture within promoters to proximal gene regulation as established by prior research” with some references added.

*“5. The INFIMA model is not unreasonable, but it is extremely densely and confusingly presented. Some clear diagrams indicating the workflow and critical dependencies would help the reader understand it more clearly.”*

**Response:**

We apologize for our inadequate explanation of the INFIMA model. We have revised the whole section “INFIMA model for fine-mapping DO mouse islet eQTLs by leveraging founder strain islet ATAC-seq and RNA-seq” and added Figs. 5d-g to illustrate an example input for the INFIMA model. We hope our explanation becomes clearer after revising.

*“5.1 I am concerned that the simulations employed to validate the results may be overfitting to the relatively small data sets.”*

**Response:**

If we are understanding the reviewer correctly, the term “overfitting” is not applicable in this setting. In our simulations, we used the real DO mice data (which is well-powered or eQTL studies as) and started from randomly simulating Z_g and V_g. Then, based on the generative model, we simulated Y_g, the DO allele effects. In the end, we fit the INFIMA model and compared the estimated Z_g and V_g with their true values. INFIMA is not a predictive model, i.e., can’t be trained on islets and applied to the liver (no notion of test and training data). Therefore, our simulation studies are designed to evaluate model performances in terms of parameter estimation. We hope this is what the reviewer was asking.

*“6. The authors presume that chromatin interactions via chromatin conformation capture are a gold standard for linking elements to genes. This is far from the case. What 'C' assays measure are proximity, and they are generally blind within 10-20kb of a TSS where. There is no indication how many variants fall into this zone.”*

**Response:**

We agree with the reviewer on the lack of specificity of using Hi-C data compared to, for example, large-scale CRISPR screening experiments. We now added a discussion on the limitations of pcHi-C data while acknowledging that this is currently a widely used approach for identifying putative links (e.g., Jung *et al*. (*Nature genetics*, 2019) utilized pcHi-C as a gold standard to identify target genes for ~27,000 GWAS SNPs and ~70,000 regulatory elements from ~2,000 human complex traits; Song *et al.* (*Nature genetics*, 2019) mapped neural cell regulatory elements to putative target genes by integrating pcHi-C, ATAC-seq, and RNA-seq; Montefiori *et al*. (*Elife*, 2018) generated pcHi-C data to link ~2,000 cardiovascular disease associated SNPs to 347 target genes.)

*“6.1 Even apart from the issue with ATAC-QTLs the claim that "INFIMA prediction of local-ATAC-QTLs for DO-eQTL genes tend to be supported by higher Hi-C interaction signal" is very weak. The significance is achieved via a highly sensitive distribution statistic (K-S); the fact that there are significant -- but quantitatively small -- differences in the aggregate is not convincing.”*

**Response:**

We agree with the reviewer that the K-S test is sensitive and this calculation warrants a closer look at the effect sizes. In order to quantify these observed differences systematically, we considered two alternatives to the K-S test and included the results in Additional file 1. First, we computed the Kullback-Leibler (KL) divergences between the empirical distributions of the Hi-C scores of the predictions from the five fine-mapping strategies as a way of quantifying the differences in these distributions (Response Table 2). We can see that, overall, INFIMA “Least Likely” is the farthest away (most dissimilar) from any of the other strategies with a total KL divergence of 1.163. This agrees well with the results that INFIMA “Least Likely” performs the worst in Fig. 7c. Similarly, INFIMA “Most Likely” strategy has the next largest KL divergence to other strategies (0.997), indicating that it is most separated from all the other strategies, followed by the “Closest to Gene” strategy (0.871).

Second, we also performed a Chi-squared test. First, we divided the normalized Hi-C scores from all strategies into 10 quantile bins (the larger the bin number, the larger normalized Hi-C score) and obtained a contingency table (Response Table 3). We can immediately see that the “Most Likely” strategy has more predictions with a higher normalized Hi-C score compared to other strategies (Response Fig. 9a). Although the strategy of choosing the SNP closest to the gene promoter (“Closest to Gene”) appears to be competitive, it is still dominated by the “Most Likely” strategy from INFIMA (Response Fig. 9b). Thus, there are visually good separations between the distributions of the five fine-mapping strategies. Response Table 4 reports the results of the Chi-squared test on pairs of rows of the contingency table in the Response Table 3 (10 tests in total) after Bonferroni correction for multiple testing. These results indicate that “Most Likely” is statistically significantly better than the remaining four fine-mapping strategies, defeating “Closest to Gene” with an adjusted p-value 3.08e-17.

Response Table 2: The Kullback-Leibler (KL) divergences between density curves of the five fine-mapping strategies.

Response Table 3: A contingency table for strategies (row) and quantile bins (column). The entry of the table represents the number of normalized Hi-C scores in the quantile bin for a strategy.

Response Fig. 9: **(a)**. A visualization of the previous contingency table (Response Table 3). **(b)**. A direct comparison between “Most Likely” and “Closest to Gene”.

Response Table 4: The Bonferroni adjusted p-values from pairwise Chi-Squared tests between distributions in 10 Hi-C score quantile bins of the five fine-mapping strategies.

*“7. The translation to human studies -- and hence support for the claim that target genes of GWAS studies can be assigned by their approach -- is highly problematic.*

*7.1 A key difficulty is presented by the translation of GWAS SNPs to the mouse genome. The overwhelming majority of GWAS SNP positions (80%) cannot be resolved directly in the mouse genome. This is expected. However, what the authors then attempt to do is to resolve these to peaks in the human genome, then resolve these peaks into the mouse genome. A priori one would have again expected a small number of correspondences. But they authors make an additional step, which not to resolve the peaks directly but to approximate them by assigning to the nearest mouse peak within 10kb. This is completely arbitrary, and not scientifically justified.*

*7.2 Associations between SNPs and human genes are conditioned on the above, and thus of concern.”*

**Response:**

We are glad that the reviewer raised this concern. We would like to highlight two points and then provide a more detailed discussion as this relates current ongoing research in our group. First, we specifically compared the pcHi-C validation rates between SNPs that directly lift over to mouse and the SNPs that lift over within the window approach. It was reassuring to see that the validation rates did not vary significantly. Second, we chose 10K to preserve the concordance between genomic compartment annotations (locations of the SNPs: intron, distal intergenic, promoter, exon,...) of the human SNPs and their lifted over locations in the mouse. However, our results (deposited to Zenodo) record the actual distances if the SNPs were lifted over indirectly by linking them to their nearest human ATAC-seq peaks. Therefore, the users can choose to not allow any indirect liftover or allow a smaller window.

We would like to offer further justification for this overall strategy from our ongoing work. Current state-of-the-art methods for identifying putative homologous regions across species (e.g. human and mouse) can be summarized as follows:

1. UCSC liftOver tool: The most common tool, based on pairwise whole genome alignments. However, as expected by the reviewer, only about ~20% of the human GWAS SNPs can be mapped to the mouse genome.
2. bnMapper (Denas *et al.*, *BMC genomics,* 2015): A method similar to UCSC liftOver using reciprocal chain files.
3. Set a small window size around a region (the window size is an interesting tuning parameter) and then apply the UCSC liftOver tool.
4. EpiAlignment (Lu *et al*., *Nucleic acids research,* 2019): A sequence and epigenome-based method using a default 20 kb window size.

We have a working manuscript named “AdaLiftOver: Genome-wide syntenic regulatory element analysis with adaptive liftOver” that rigorously compares these approaches and explicitly addresses the concerns raised by the reviewer. In our AdaLiftOver paper, we treat the 10 kb distance as a tuning parameter. There are 3 major steps of AdaLiftOver:

1. Direct liftOver (d): We directly apply the UCSC liftOver tool for a set of human GWAS SNPs (1 bp regions).
2. Cis liftOver (c): If we are unable to identify the GWAS orthologous regions in mouse using direct liftOver, we search for nearby regulatory elements (e.g. ATAC-seq peaks, ChIP-seq peaks) within a window (a tuning parameter, in this case 10 kb). We then map these regulatory elements using UCSC liftOver instead.
3. Local liftOver (l): For the GWAS regions that fail to map in steps 1 & 2, we increment the local window size (step size is 50 bp by default) up to a threshold (500 bp by default) until the regions are mappable using UCSC liftOver tool.

We call our Adaptive liftOver using the first 2 steps and all 3 steps “AdaLiftOver (dc)” and “AdaLiftOver (dcl)”, respectively. We note that the AdaLiftOver (dc) is the same method we used in the section “INFIMA generates candidate susceptibility genes for human GWAS SNPs”. We benchmarked these two AdaLiftOver methods using homologous human and mouse ATAC-seq data from blood cells (B, CD4, CD8, CMP, ERY, GMP, Mega, MEP, Mono, NK). We applied each mapping method to human GWAS SNPs from 5 traits (3 traits related to blood: MCV, MPV, LYMPH_COUNT; 2 traits treated as controls: Alzheimer, Type2Diabetes). For the mapped regions in the mouse, we then utilized bootstrapping and estimated the proportion of their overlaps with mouse ATAC-seq peaks (Response Fig. 10). The higher rate of overlapping with blood-related regulatory regions, the better the mapping method is. As expected, the overlapping proportions are equally low for all methods for control traits thus there does not appear to be biases that influence our conclusion. For blood traits, we observe that AdaLiftOver (dc) performs better than existing methods including the recent epigenome-based approach “EpiAlignment” (Response Fig. 10). In addition, the commonly used “local liftOver” strategy may not provide meaningful mapping results.

Response Fig. 10: Benchmarking AdaLiftOver methods against traditional mapping approaches. X-axis: Alzheimer and Type2 Diabetes are treated as control groups while MCV, MPV, and LYMPH_COUNT are blood related traits for benchmarking. Y-axis: the proportion of the mouse regions, which can be mapped using a specific method, that overlap with mouse ATAC-seq peaks in blood. The higher the better.

*“8. Fig S18 suggests that Cast is driving much of their signal -- which highlights the fact that strain biases are not well accounted for.”*

**Response:**

CAST is a wild strain which is quite different from the reference strain B6. DO mice study exploits the large degree of genetic variation contributed by CAST to link genetic differences with physiological differences. We understand that if we wanted to draw conclusions for other factors, e.g. sex effects, we would have to control for the strain differences. In our case, however, the strain effects are major parameters of interest.

*“8.1 It is also not clear to me how one can get a negative abs log p value; how does one understand a p value greater than 1?”*

**Response:**

We could not locate where a p value larger than 1 is listed. Perhaps our log transformation and effect size indication is leading to a problem. Below are all the plots with a -log10(p-value) axis.Here, P-value <= 1 implies -log10(p-value) >= 0.

Figure 3c: The size of each dot denotes “-log10(pval_rank)” where pval_rank measures the significance of atSNP results for in-silico mutation analysis.

Figure 8b: The y-axis “-log10(adj.pval)” ranges from 0 to 6+. This figure demonstrates enrichment results for local-ATAC-QTL and GWAS SNPs for traits.

Figure S21: The x-axis is “|-log10(pval_fpd)|” while the y-axis is “|-log10(pval_rank)| ”. The absolute values are the typical “minus log10 p-values” to represent statistical significance. Although there are negative portions of both x-axis and y-axis, it does not mean that we have p-values > 1. Instead, we explained the meaning of negative signs in the caption. Negative values in the x-axis means \Delta FPD < 0 (footprint disruption) while positive values means \Delta FPD > 0 (footprint enhancing). Negative values in the y-axis means motif disruption while positive values means motif enhancing. We used this figure to demonstrate how we obtained individual footprints with concordant footprint changes and motif changes.

We think that perhaps Figure S21 led to a confusion. We have modified the the x-axis label from “|-log10(pval_fpd)|” to “|-log10(pval_fpd)|*sign(\Delta FPD)” and the y-axis label from “|-log10(pval_rank)|*(-1)^I{motif disruption}”. We hope that Figure S21 is clearer now.

*“9. On a general level, I found many of the figures to be confusing, lacking adequate explanation in the legends. Virtually none of them were what I would consider self-explanatory.”*

**Response:**

We apologize for not making every legend/label clear enough. We hope our manuscript becomes clearer after a significant amount of revision. We would like to thank the reviewer for taking the time and helping us improve it.

References:

Baek, S., Goldstein, I., & Hager, G. L. (2017). Bivariate genomic footprinting detects changes in transcription factor activity. *Cell reports*, *19*(8), 1710-1722.

Broman, K. W., Gatti, D. M., Simecek, P., Furlotte, N. A., Prins, P., Sen, Ś., ... & Churchill, G. A. (2019). R/qtl2: software for mapping quantitative trait loci with high-dimensional data and multiparent populations. *Genetics*, *211*(2), 495-502.

Denas, O., Sandstrom, R., Cheng, Y., Beal, K., Herrero, J., Hardison, R. C., & Taylor, J. (2015). Genome-wide comparative analysis reveals human-mouse regulatory landscape and evolution. *BMC genomics*, *16*(1), 1-9.

Grubert, F., Zaugg, J. B., Kasowski, M., Ursu, O., Spacek, D. V., Martin, A. R., ... & Snyder, M. (2015). Genetic control of chromatin states in humans involves local and distal chromosomal interactions. *Cell*, *162*(5), 1051-1065.

Gusmao, E. G., Allhoff, M., Zenke, M., & Costa, I. G. (2016). Analysis of computational footprinting methods for DNase sequencing experiments. *Nature methods*, *13*(4), 303-309.

Jung, I., Schmitt, A., Diao, Y., Lee, A. J., Liu, T., Yang, D., ... & Ren, B. (2019). A compendium of promoter-centered long-range chromatin interactions in the human genome. *Nature genetics*, *51*(10), 1442-1449.

Lu, J., Cao, X., & Zhong, S. (2019). EpiAlignment: alignment with both DNA sequence and epigenomic data. *Nucleic acids research*, *47*(W1), W11-W19.

Montefiori, L. E., Sobreira, D. R., Sakabe, N. J., Aneas, I., Joslin, A. C., Hansen, G. T., ... & Nóbrega, M. A. (2018). A promoter interaction map for cardiovascular disease genetics. *Elife*, *7*, e35788.

Quach, B., & Furey, T. S. (2017). DeFCoM: analysis and modeling of transcription factor binding sites using a motif-centric genomic footprinter. *Bioinformatics*, *33*(7), 956-963.

Sherwood, R. I., Hashimoto, T., O'donnell, C. W., Lewis, S., Barkal, A. A., Van Hoff, J. P., ... & Gifford, D. K. (2014). Discovery of directional and nondirectional pioneer transcription factors by modeling DNase profile magnitude and shape. *Nature biotechnology*, *32*(2), 171-178.

Song, M., Yang, X., Ren, X., Maliskova, L., Li, B., Jones, I. R., ... & Shen, Y. (2019). Mapping cis-regulatory chromatin contacts in neural cells links neuropsychiatric disorder risk variants to target genes. *Nature genetics*, *51*(8), 1252-1262.

Yardimci GG, Frank CL, Crawford GE, Ohler U. Explicit DNase sequence bias modeling enables high-resolution transcription factor footprint detection. Nucleic Acids Res. 2014;42:11865–78.

Karabacak Calviello, A., Hirsekorn, A., Wurmus, R. *et al.* Reproducible inference of transcription factor footprints in ATAC-seq and DNase-seq datasets using protocol-specific bias modeling. *Genome Biology* 20, 42 (2019). https://doi.org/10.1186/s13059-019-1654-y.

**Second round of review**

**Reviewer 1**

The authors have addressed my comments.
